# The potential role of phentermine in the treatment of obesity in the adult population of Mexico

**DOI:** 10.3389/fmed.2026.1912418

**Published:** 2026-10-08

**Authors:** Abraham I. Villarreal-Castellanos, Héctor I. Rocha-González, María E. Sánchez-Mendoza, Jesús Arrieta-Valencia, Ana L. Pardo-Gutiérrez, Juan Rodríguez-Silverio, Juan C. Huerta-Cruz, José E. Roa-Coria, Lina M. Barranco-Garduño, Juan G. Reyes-García

**Affiliations:** 1 Sección de Estudios de Posgrado e Investigación, Escuela Superior de Medicina, Instituto Politécnico Nacional, Mexico City, Mexico; 2 Laboratorio de Farmacología Clínica y Experimental, Instituto Nacional de Enfermedades Respiratorias, Ismael Cosío Villegas, Mexico City, Mexico

**Keywords:** body weight reduction, efficacy, obesity, phentermine, predictor factors, tolerance

## Abstract

**Background:**

The recently published Mexican guidelines for the management of obesity recommend the alternative use of phentermine for six months or more in adults, based on its low cost and wide availability. However, despite the extensive clinical use of phentermine, accurate information remains limited regarding the clinical appropriateness of selecting a 15 mg versus a 30 mg starting dose, as well as their respective efficacy expectations. This study was aimed to advance these aspects, based in the first month body weight reduction in kilograms (1mo-BWRkg) and month of tolerance development (moT) as efficacy predictors of 15 mg and 30 mg phentermine, on obese Mexicans.

**Methods:**

A comparative, prospective, multi-center, uncontrolled phase IV, open-label study was performed in 114 and 151 obese completer subjects treated for 6 months with daily 15 mg and 30 mg phentermine, respectively, between August 2015 and December 2018. Subjects were grouped into various 1mo-BWRkg, moT and 6mo-BWR% (six-month body weight reduction in percentage) intervals. Correlations between 1mo-BWRkg, moT and 1mo-BWRkg + moT with 6mo-BWR% efficacy and a multivariate analysis was performed to determine the impact of these parameters on the 6mo-BWR% efficacy with both phentermine doses.

**Results:**

The BWR% profile of 30 mg phentermine was only marginally superior to that of 15 mg phentermine. A large intrasubject variability in BWR% was observed with both interventions. 1mo-BWRkg + moT was found to be an acceptable parameter of 6mo-BWR% for 15 mg and 30 mg phentermine.

**Conclusion:**

Our results suggest that once phentermine is selected for initial obesity treatment, starting with a 15 mg dose is a rational approach. An advantage of phentermine is that its 6mo-BWR% effectiveness can be predicted early and progressively. Furthermore, phentermine seems to be a viable long-term alternative to the newer, highly effective, and costly weight-loss drugs for acquisition of healthy lifestyle habits, the improvement of quality of life and life expectancy, and the prevention of health risks in the Mexican population, but requires confirmatory prospective controlled studies.

## Introduction

Beyond its definition as the abnormal or excessive accumulation of fat that poses a health risk (1), obesity may be empirically described as a complex, multifactorial, recurrent, chronic, and highly prevalent condition that is associated with a reduced life expectancy and an elevated risk of numerous diseases (2, 3). The complexity of obesity is comparable to the comprehension of its treatment, which is based on variable body weight reduction (BWR) objectives that are not necessarily linked to the restoration of normal weight (4, 5). The clinical objectives of managing obesity are varied and include the acquisition of healthy lifestyle habits, the prevention of health risks, the reversal of comorbidities, and the improvement of quality of life and life expectancy (6), which contrast with the cosmetic objectives related to high expectations of BWR (7–9). The experience with current options for weight management, indicates that the efficacy and risks of interventions, whether lifestyle modification, pharmacotherapy and bariatric surgery, increase in that same order, yet in inverse proportion to their availability. Furthermore, all of these modalities are characterized by significant interindividual variability in the magnitude of BWR achieved (10).

Although behavioral modification through diet and exercise is the fundamental approach to obesity treatment, a substantial number of obese patients fail to achieve a meaningful reduction in body weight with this modality (11). Thus, the early rational justification for the use of drugs has been to maintain adherence to the prescribed healthy changes. Subsequently, the finding that the sustained reduction of body weight of 5%–10% produces significant clinical benefits led to the consideration of this figure as a basic element for approving the minimum level of efficacy of the new anti-obesity medications (AOMs) (5, 12).

In general, the weight loss response induced by short- or long-term AOMs is highly variable and may range from no weight loss to reductions of 20% or more over the course of treatment; however, these reductions are not necessarily sustained indefinitely (13, 14). The availability of pharmacological resources for the treatment of obesity is really limited and varies from country to country depending on the policies of the corresponding regulatory bodies. The newer, highly effective, and costly AOMs tirzepatide and semaglutide, are available in Mexico, in coexistence with low-cost older AOMs like orlistat and phentermine. Some authors have pointed out the fact that newer AOMs are not always inherently better or more effective for a given patient than an older one, since the individual heterogeneity in response to any of these drugs (15).

In this regard, the Mexican clinical practice guidelines for the management of the highly prevalent problem of overweight and obesity were recently published. The guidelines recommend considering the use of the old drug, previously marginalized phentermine, for a period of six months or more in adults aged 18 years or older who may benefit from such treatment based on its low-cost and wide availability, which may be especially useful for patients with lower income and those who lack access to the newer, costlier medications (16). In addition, and unexpectedly, a clinical protocol evaluating the long-term use of submaximal doses (24 mg) of phentermine for two years in US-American, with the aim of providing evidence to support the use of this low-cost option is being carried out (17).

In Mexico, several short-term anorexigenic drugs are available, including various concentrations of phentermine (8 mg, 15 mg and 30 mg), as well as immediate- and extended-release formulations of this drug for oral administration. However, there are no published clear guidelines for selecting the appropriate initial concentration, the suitability of dose titration, or for determining the expectations of the corresponding level of efficacy. The purpose of this study was to advance in these aspects, based on the analysis of several previous findings from mexican population studies, including an exploratory study on the comparative efficacy of 15 mg and 30 mg doses of phentermine, and another on the first month body weight reduction in kilograms (1mo-BWRkg) and month of tolerance development (moT) as efficacy predictors of 30 mg phentermine.

## Material and methods

The current study was performed as a per-protocol analysis. The efficacy of oral administration of 15 mg or 30 mg phentermine assessed in a comparative, prospective, multi-center, uncontrolled phase IV, open-label study was analyzed. A sample of 265 Mexican subjects with a body mass index (BMI) ≥ 30 kg/m^2^ and an age of 18 years or older who had completed 6 months of treatment from 36 clinics situated in 12 states of the Mexican Republic between August 2015 and December 2018 were considered. Originally, 400 and 532 subjects were arbitrarily assigned to the 15 mg and 30 mg phentermine doses, respectively. Based on available prescribing information, exclusion criteria were hypersensitivity to sympathomimetic drugs, use of other AOMs, use of monoamine oxidase inhibitors, psychiatric disorders, renal diseases, closed-angle glaucoma, coronary artery disease, stroke, arrhythmias, congestive heart failure, valvulopathies, history of substance abuse, and pregnant or breast-feeding women.

In the first visit, medical history and clinical examination were employed to ascertain the baseline health status of patients, where some characteristics including history of smoking, alcohol consumption and sedentary habits were explored. Patients were required to empty their bladder upon arrival, dress with a hospital gown, and a body in underwear was weighed with a calibrated scale, height was determined with the patient standing with the heels together, and the buttocks, shoulders, and head in contact with the stadiometer. Systolic and diastolic blood pressure were determined with an electronic sphygmomanometer. To measure fasting plasma glucose, a blood sample was taken and analyzed by standard laboratory blood chemistry measurements. Patients were arbitrarily allocated to receive 15 mg phentermine or 30 mg phentermine capsules (Terfamex®), which were prescribed to be taken orally, once every morning before breakfast, for 6 months. All capsules were provided by Productos Medix, S.A. de C.V. (Mexico City, Mexico). Patients were instructed to follow a diet of 1500 kcal/day, for which various menus were suggested, and to carry out physical activity for 20 min of walking daily.

At every visit, percentage body weight reduction (BWR%) was calculated with the proportion of weight lost for each month with respect to baseline weight, whereas the body weight reduction in kilograms (BWRkg) was calculated by subtracting the weight at the beginning of the study from weight at any other given evaluation time in the six-month study period. Patients were grouped according to their first-month body weight reduction in kilograms (1mo-BWRkg) as follows: <1 kg, 1 to <2 kg, 2 to <3 kg, 3 to < 4 kg, 4 to <5 kg, 5 to <6 kg, 6 to <7 kg, 7 to <8 kg, 8 to <9 kg, 9 to <10 kg, and ≥10 kg. Tolerance to the weight-reducing effect of phentermine was defined as the absence of body weight loss for any given month with respect to the prior month (moT). Consequently, patients were classified into 6 groups according to the month when they developed tolerance to treatment (2moT, 3moT, 4moT, 5moT and 6moT) or if tolerance did not occur (NT). These groups were further categorized into ranges of 1mo-BWRkg <3 kg, 3 to <6 kg or ≥6 kg and tolerance development at the 2nd and 3rd months (2-3 moT or early tolerance), 4th and 5th months (4-5 moT or late tolerance) or 6th month and no tolerant (6moT-NT or no tolerant),

Correlations between 1mo-BWRkg, moT and 1mo-BWRkg + moT with 6mo-BWR% efficacy were calculated using Pearson's correlation test to determine the impact of these parameters on the 6mo-BWR% efficacy. Treatment efficacy was evaluated with the non-cumulative percentage of patients who achieved a BWR% of 5% to <10%, 10% to < 15%, 15% to <20% or ≥20%. In addition, a multivariate analysis was performed to investigate simultaneously 1mo-BWRkg + moT, age, basal weight, sex, glucose level, and treatment on 6mo-BWR%. Based on the effect of the 2 priorly analyzed variables on the efficacy of treatments, 1mo-BWRkg groups were divided into subgroups, according to the development of early tolerance at month 2 and 3 (2-3 moT), late tolerance (4-5 moT) and tolerance at 6 months or not tolerant (6moT-NT) for qualitative analysis (Figure 1). Differences were considered statistically significant, with a bilateral *p* < 0.05. Statistical analyses were carried out in the GraphPad Prism v.6.0 software and Jamovi v2.7 software.

**Figure 1:**
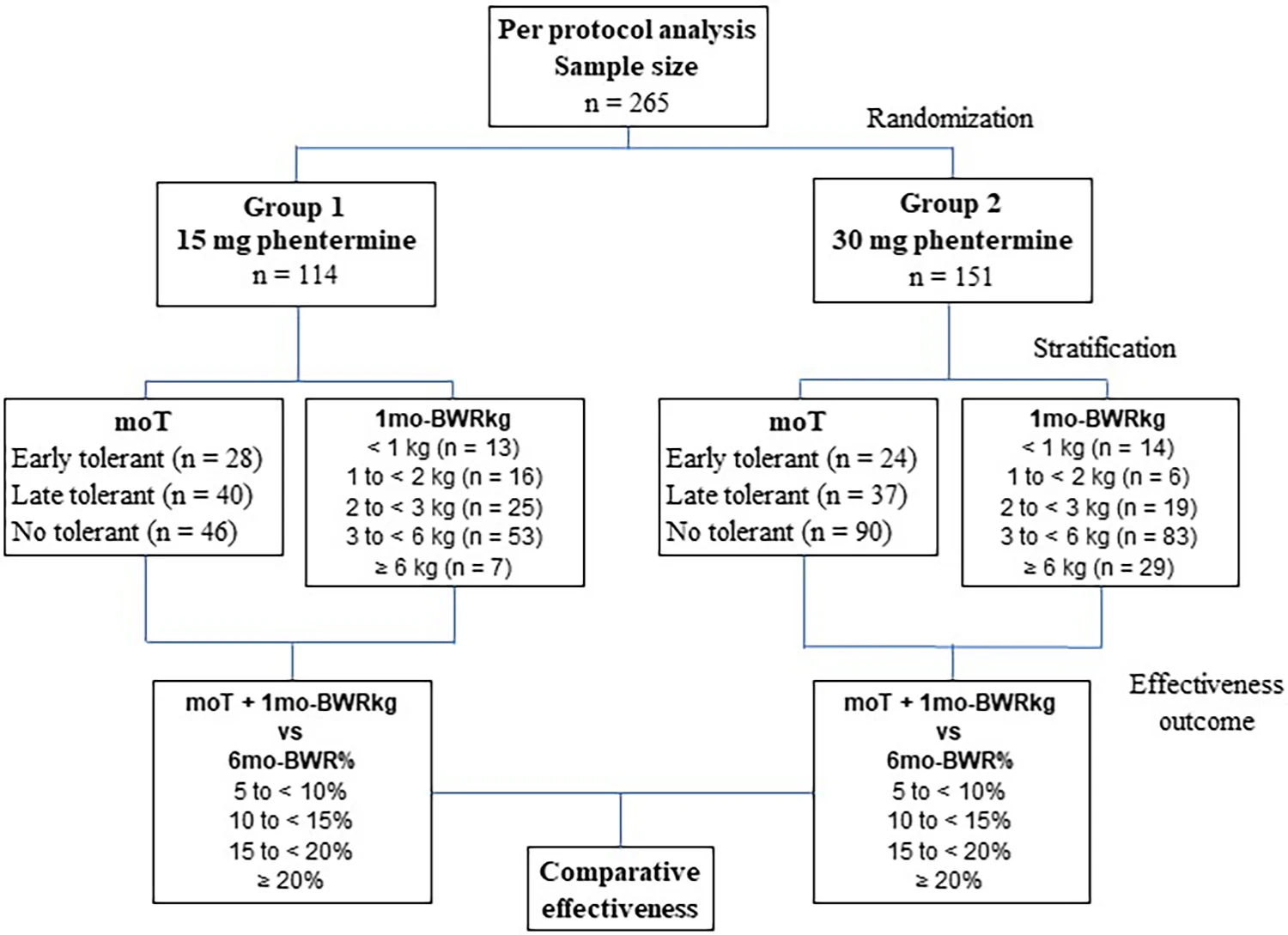
Flow chart of the study.

## Results

### Demographic data

The demographic variables were balanced between 15 mg and 30 mg phentermine groups. Nearly 80% of patients treated with 15 mg (114 subjects) or 30 mg of phentermine (151 subjects) were women around 40 years of age who had mainly class I obesity. Participants reported being mostly non-smokers and alcohol non-drinkers, although they were sedentary. The proportions of patients with prediabetic or diabetic fasting plasma glucose levels were not significantly different between the groups. The proportion of hypertensive patients was significantly higher in the 15 mg phentermine group (Table 1).

**Table 1:** Baseline demographic characteristics.

Parameter	Phentermine	
	15 mg (*n* = 114)	30 mg (*n* = 151)
Sex^b^		
Female	95 (83.3)	120 (79.4)
Male	19 (16.7)	31 (20.6)
Age (years)^a^	42.9 (13.6)	40.1 (12.6)
BMI (kg/m^2^)^a^		
30–34.9	83 (72.8)	100 (66.2)
35–39.9	22 (19.2)	43 (28.5)
≥ 40	9 (7.8)	8 (5.3)
FPG (mg/dL)^a^		
> 99 - < 125	40 (35.0)	49 (32.4)
≥ 125	15 (13.1)	10 (6.6)
Hypertension^b^	9 (7.8) *	3 (0.9)
Sedentary lifestyle^b^	108 (94.7)	142 (94.0)
Smokers^b^	6 (5.2)	7 (4.6)
Daily alcohol consumer^b^	0 (0)	0 (0)

Data are expressed as ^a^ mean (SD) or as ^b^ frequencies (%). FPG: fasting plasma glucose; BMI: body mass index; SD: standard deviation. *Significantly different by Student's t-test or Fisher's test (*p* < 0.05).

### Efficacy profile of 15 and 30 mg phentermine

The time courses of BWR% induced by the administration of 15 mg and 30 mg phentermine were similar, with 6mo-BWR% of 9.3% and 10.4%, respectively (Figure 2A). Also, a comparable proportion of subjects treated with 15 or 30 mg phentermine achieved 6mo-BWR% in the intervals of 5% to < 10% (29.8% vs. 24.5%), 10% to < 15%, (34.2% vs. 42.3%) and 15% to < 20% (10.5% vs. 12.5%) (**not shown in graph**), with no significant differences, except at the > 20% level (1.7% vs. 7.2%, respectively, Fisheŕs test). Additionally, typical and large intrasubject variability was observed at all time points with both interventions **(**Figure 2B).

**Figure 2:**
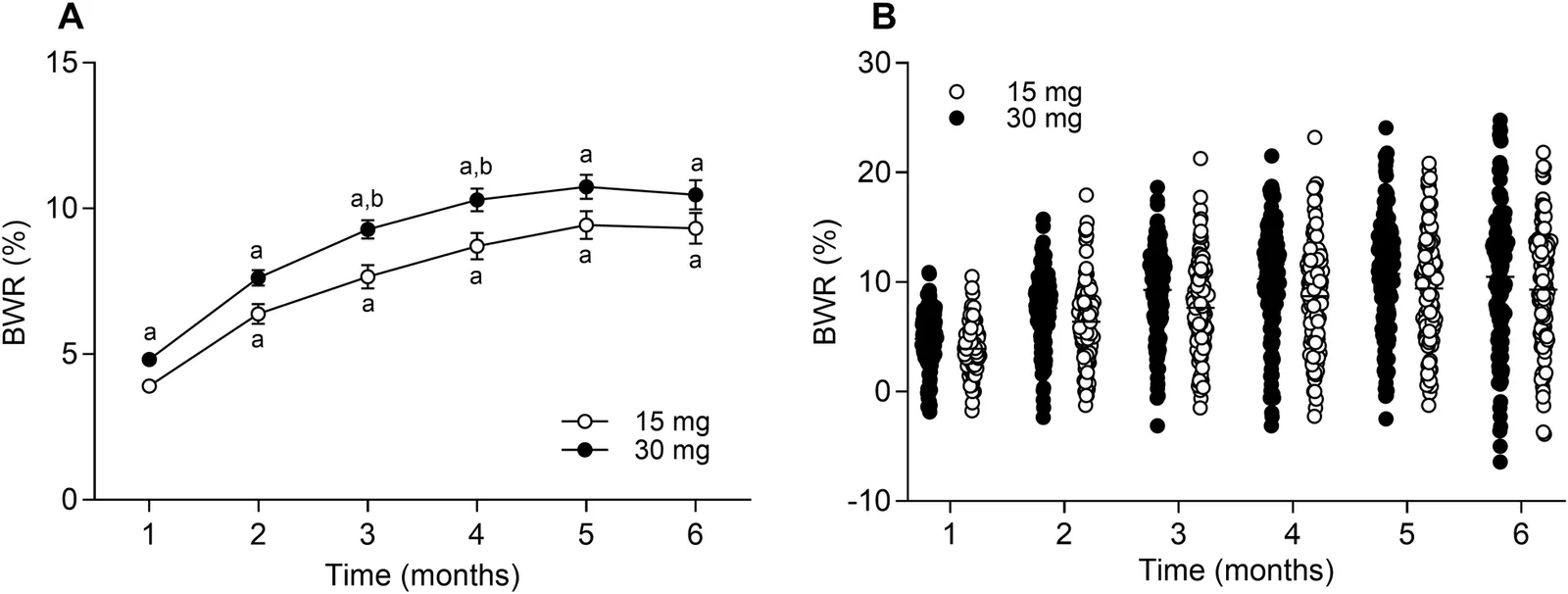
Time course of percentage of body weight reduction (BWR%) of subjects treated with 15 mg (*n* = 114) or 30 mg (*n* = 151) phentermine**.** Panel **(A).** Each point represents the mean BWR% (± SEM). ^a^ Significantly different from basal point and ^b^ significantly different intergroup for each time point by two-way ANOVA followed by Tukeýs test (*p* < 0.05). Panel **(B).** Each point represents individual data.

### 1mo-BWRkg induced by 15 mg and 30 mg phentermine

The proportion of subjects treated with 15 mg phentermine tended to be greater, similar, and less at lower, intermediate, and higher 1mo-BWRkg levels, respectively, than 30 mg phentermine, Figure 3A.

**Figure 3:**
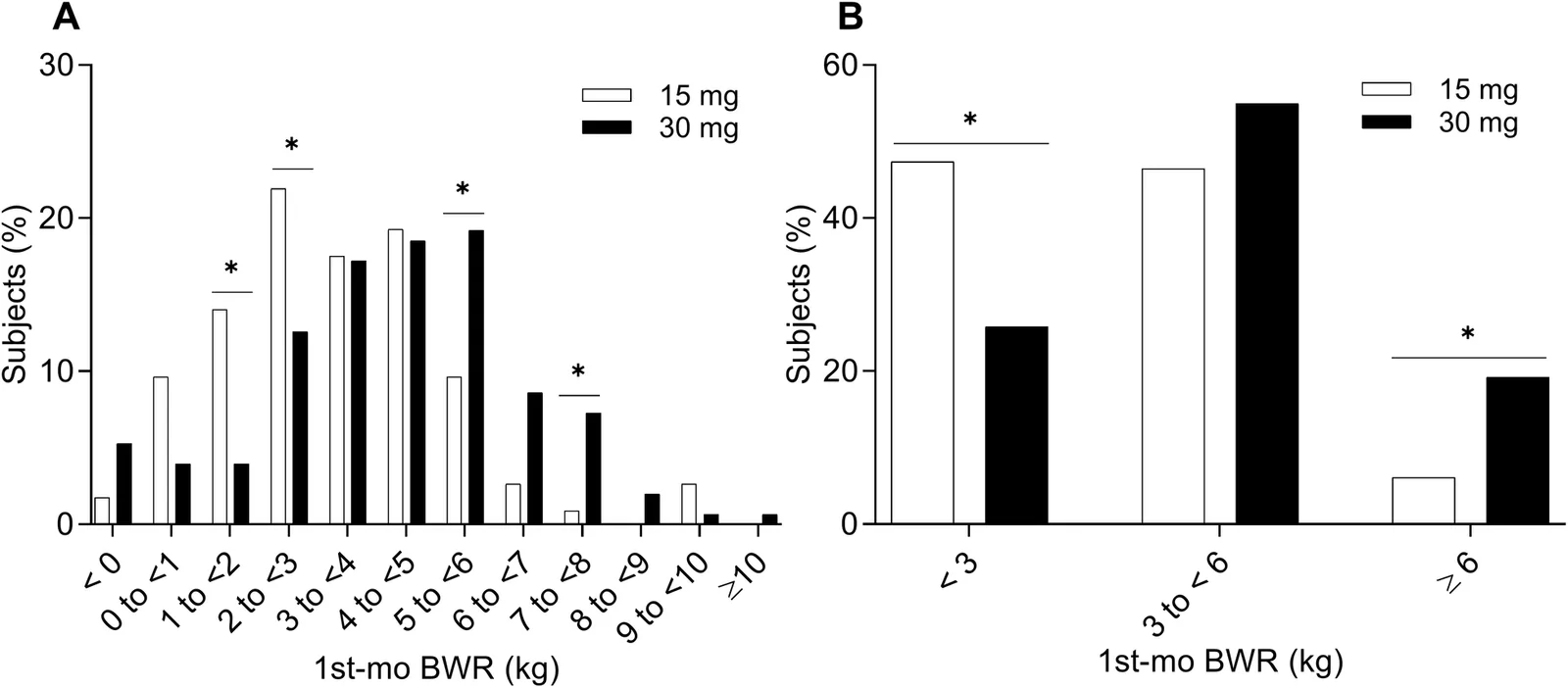
First-month body weight reduction in kilograms (1mo-BWRkg) induced by the administration of 15 mg (*n* = 114) and 30 mg (*n* = 151) phentermine. Panel **(A)**. Each bar represents the percentage of subjects who achieved a 1mo-BWRkg in increasing unit intervals. Panel **(B)**. Each bar represents the percentage of subjects who achieved a 1mo-BWRkg in grouped relevant increasing unit intervals. *Significantly different by Fisher's test (*p* < 0.05).

Statistically significant differences favoring 15 mg phentermine were found in the 1mo-BWRkg range of < 3 kg (47.3% vs. 25.8% of 30 mg phentermine) and in favor of 30 mg phentermine in the 1mo-BWRkg range ≥ 6 kg (19.2% vs. 6.1% of 15 mg phentermine), but not in the intermediate 1mo-BWRkg range of 3 to < 6 kg (46.4% vs. 54.9% of 15 mg phentermine vs. 30 mg phentermine), Figure 3B.

### Tolerance development to 15 mg or 30 mg phentermine

In contrast to what was observed in the 1mo-BWRkg profile, the proportion of subjects considered tolerant did not appear to follow a distinctive monthly pattern, and the grouping of these into early tolerant (2 to 3 months: 24.5% vs. 23.0%), late tolerant (4 to 5 months: 35.0% vs. 38.8%) or non-tolerant (at the end of 6 months of treatment or truly non-tolerant NT: 40.3% vs. 38.1%) did not exhibit statistically significant differences between 15 mg phentermine and 30 mg phentermine interventions, respectively, Figure 4.

**Figure 4:**
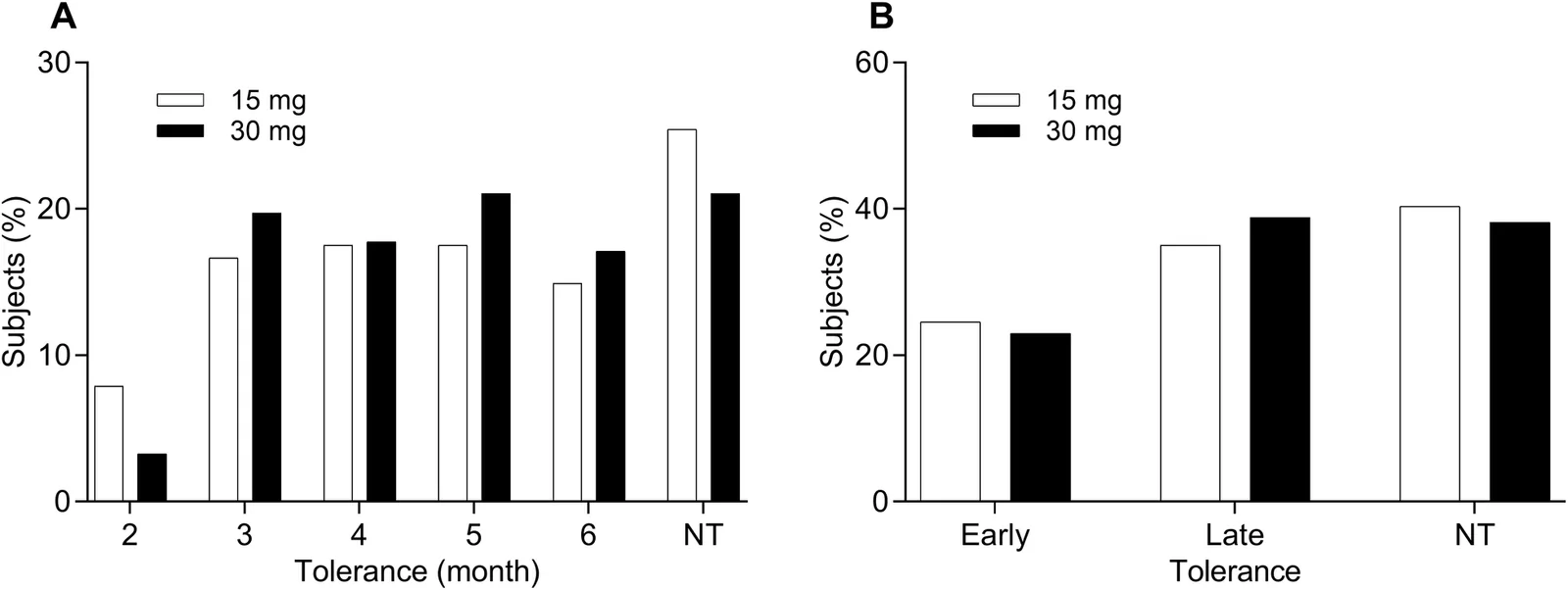
Month of tolerance (moT) or no tolerance (NT) related with the administration of 15 mg (*n* = 114) or 30 mg (*n* = 151) phentermine. Panel **(A)**. Each bar represents the corresponding percentage of subjects. Panel **(B)**. Each bar represents the percentage of subjects grouped in relevant increasing early (2-3 moT), late (4-5 moT) or NT (6 moT and NT) intervals. There is no significant difference by Fisher's test.

### Correlations of 1mo-BWRkg, moT, and 1mo-BWRkg + moT for 6mo-BWR% as efficacy predictors

The single correlation analysis of 1mo-BWRkg or moT vs. 6mo-BWR% revealed acceptable and significant Pearson r coefficients ranging around 0.5 in both phentermine groups (15 mg or 30 mg), Table 2. Interestingly, the correlation increased to a 0.774 (*p* < 0.001) level for 15 mg phentermine and 0.652 (*p* < 0.001) for 30 mg phentermine when combining 1mo-BWRkg + moT vs. 6mo-BWR%, Figure 5.

**Table 2:** Correlations of 1mo-BWRkg and moT, with 6mo-BWR%.

Sets of data	Phentermine	
	15 mg	30 mg
1mo-BWRkg vs. 6mo-BWR%	r = 0.64*	r = 0.46*
moT vs. 6mo-BWR%	r = 0.50*	r = 0.56*

Data are presented as Pearson correlation coefficients (r) for first-month body weight reduction in kilograms (1mo-BWRkg) or month of tolerance development (moT) for six-month body weight reduction in percentage (6mo-BWR%). *Statistically significant with a *p* < 0.05.

**Figure 5:**
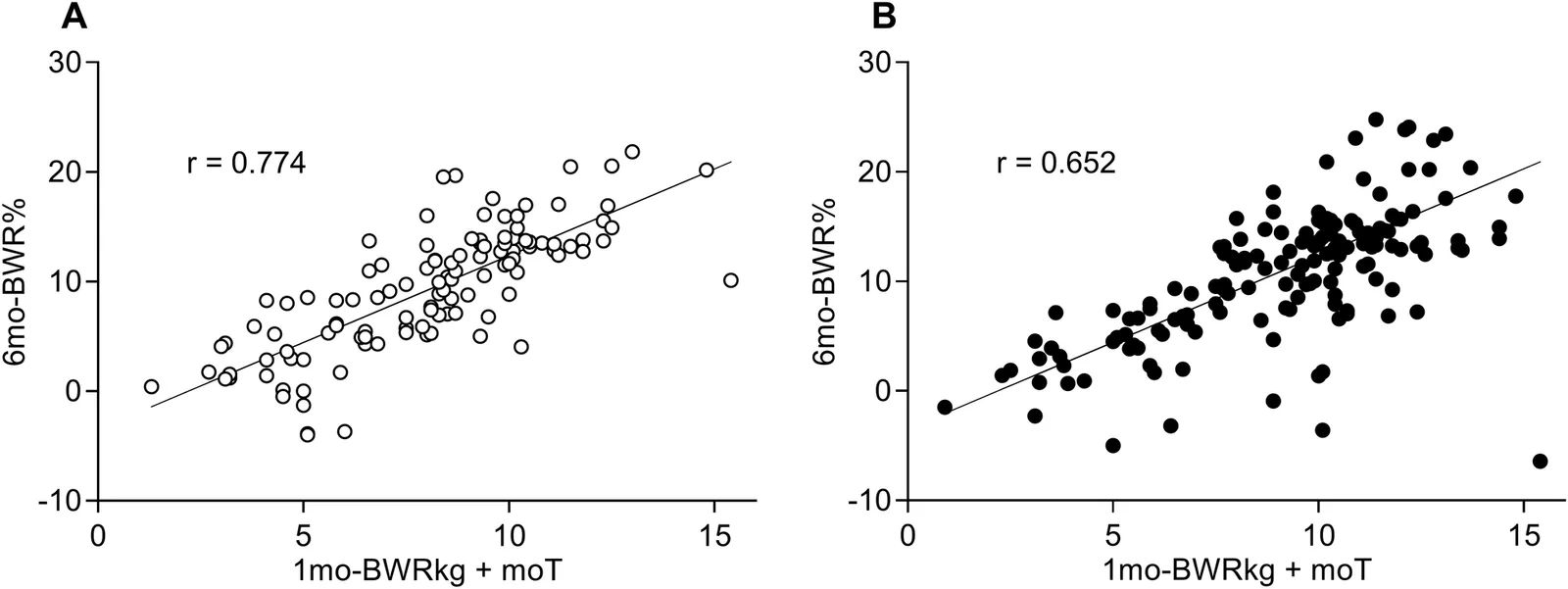
Data are presented as Pearson correlation coefficients **(r)** for first-month body weight reduction in kilograms (1mo-BWRkg) and month of tolerance development (moT) versus six-month body weight reduction in percentage (6mo-BWR%) for phentermine. Panel **(A)**. 1mo-BWRkg and moT versus 6mo-BWR% for 15 mg phentermine. Panel **(B)**. 1mo-BWRkg and moT versus 6mo-BWR% for 30 mg phentermine. *Statistically significant with a *p* < 0.05.

The multivariate model produced a R^2^ of 0.526 where 1mo-BWRkg + moT (*β* = 1.4864, SE = 0.0927, *p* < 0.001); but not age (*β* = −0.0244, SE = 0.0232, *p* = 0.293), sex (*β* = −1.1413, SE = 0.7874, *p* = 0.148), glucose level (*β* = −0.0125, SE = 0.0132, *p* = 0.347) or treatment (*β* = −0.2463, SE = 0.5747, *p* = 0.669), was significantly related to 6mo-BWR%. Basal weight was also significantly related to 6mo-BWR% (*β* = −0.0555, SE = 0.0256, *p* = 0.031) but to a lesser grade.

### Impact of 1mo-BWRkg + moT on the 6mo-BWR% efficacy induced by 15 mg and 30 mg phentermine

Table 3 shows the proportion of subjects per treatment group who achieved 6mo-BWR% in the intervals 5 to < 10%, 10 to < 15%, 15 to < 20% or ≥ 20%, stratified by 1mo-BWRkg in the intervals < 1 kg, 1 to < 2 kg, 2 to < 3 kg, 3 to < 6 kg and ≥ 6 kg; and moT in the intervals 2-3 moT, 4-5 moT, and 6moT-NT. With the intention of defining some generalizations, the different 1mo-BWRkg + moT subgroups were highlighted in white, light green or dark green, according to the considered increasing efficacy of the 6mo-BWR%. In this way, the subgroups in the 1mo-BWRkg < 1 kg, 1 to < 2 kg and 2 to < 3 kg in early tolerant subjects; and < 1 kg and 1 to < 2 kg in late tolerant and no tolerant subjects, showed 6mo-BWR% effectiveness of up to 5 to 10%. In addition, the subgroups in the 1mo-BWRkg 3 to <6 kg in early tolerant subjects, and 2 to 3 kg in late tolerant and no tolerant subjects, tended to show mainly 6mo-BWR% of 5 to <10% and a lower percentage of 10 to < 15%. Finally, the subgroups in the 1mo-BWRkg ≥6 kg in early tolerant subjects, and 3 to <6 kg and ≥6 kg in late tolerant and no tolerant subjects, primarily lean towards a 6mo-BWR% of 5 to <10% with some cases of 15 to < 20% or ≥ 20%.

**Table 3:** Six-month body weight reduction in percentage (6mo-BWR%) induced by 15 and 30 mg phentermine according to first-month body weight reduction in kilograms (1mo-BWRkg) and month tolerance development (moT).

15 mg Phentermine (*n* = 114)				30 mg Phentermine (*n* = 151)			
1mo-BWRkg	*n*	6mo-BWR%	Efficacy *n* (%)	1mo-BWRkg	*n*	6mo-BWR%	Efficacy *n* (%)
Early tolerant (2-3 moT)							
<1	4	5 to <10	1 (25)	<1	7	5 to <10	–
1 to <2	8	5 to <10	1 (13)	1 to <2	2	5 to <10	–
2 to <3	6	5 to <10	6 (100)	2 to <3	5	5 to <10	3 (60)
3 to <6	9	5 to <10	6 (67)	3 to <6	10	5 to <10	6 (60)
		10 to <15	2 (22)			10 to <15	2 (20)
≥6	1	15 to <20	1 (100)	≥6	–	–	–
Late tolerant (4-5 moT)							
<1	6	5 to <10	–	<1	2	5 to <10	–
1 to <2	6	10 to <15	1 (17)	1 to <2	4	10 to <15	–
2 to <3	5	5 to <10	2 (40)	2 to <3	5	5 to <10	3 (60)
		10 to <15	2 (40)			10 to <15	–
3 to <6	19	5 to <10	6 (32)	3 to <6	16	5 to <10	3 (19)
		10 to <15	8 (42)			10 to <15	10 (62)
		15 to <20	4 (21)			15 to <20	3 (19)
≥6	4	5 to <10	–	≥6	10	5 to <10	3 (30)
		10 to <15	3 (75)			10 to <15	4 (40)
		15 to <20	1 (25)			15 to <20	2 (20)
No tolerant (6 month-NT)							
<1	3	5 to <10	1 (33)	<1	5	5 to <10	1 (20)
1 to <2	2	5 to <10	2 (100)	1 to <2	–	5 to <10	–
2 to <3	14	5 to <10	7 (50)	2 to <3	9	5 to <10	5 (56)
		10 to <15	5 (36)			10 to <15	4 (44)
		15 to <20	1 (7)			15 to <20	–
3 to <6	25	5 to <10	2 (8)	3 to <6	57	5 to <10	11 (19)
		10 to <15	17 (68)			10 to <15	23 (40)
		15 to <20	4 (16)			15 to <20	11 (19)
		≥20	–			≥20	8 (14)
≥6	2	5 to <10	–	≥6	19	5 to <10	2 (11)
		10 to <15	1 (50)			10 to <15	11 (58)
		15 to <20	–			15 to <20	3 (16)
		≥20	1 (50)			≥20	3 (16)

Data are expressed as the non-cumulative number of subjects (%) who achieved several levels of 6mo-BWR% (5 to <10%, 10 to <15%, 15% to <20%, and ≥20%), according 1mo-BWRkg (<1 kg, 1 to <2 kg, 2 to <3 kg, 3 to <6 kg or ≥6 g) and moT (early: 1-2 moT; late: 3-4 moT or no tolerant: 6 moT-NT). Subgroups 1mo-BWRkg + moT was highlighted in white, light green, or dark green, according to the consideration of 6mo-BWR% effectiveness.

## Discussion

The prevalence of overweight and obesity in Mexico has increased significantly in recent decades. According to BMI data, it is estimated that currently, 75.2% of Mexican adults over 20 years of age suffer from overweight (38.3%) and some degree of class 1 (24.2%), class 2 (8.7%) or class 3 obesity (4.0%) (18).

The recently published Mexican clinical practice guidelines for the management of overweight and obesity include a paradigmatic shift, following the recommendation of the use of the traditionally marginalized drug phentermine as monotherapy for six months or more and the implicit acceptance of a role for short-term medications in adult obesity therapy (19). Due to a lack of precise guidelines on the appropriate use and expected efficacy of the most common strength doses of phentermine, the objective of this analysis was to delve deeper into these aspects. This study was based on the sub-analysis of a sample of participants who completed six months of a previous study under real-life conditions using 15 mg or 30 mg phentermine, including the 6mo-BWR% at according to the 1mo-BWRkg and moT.

This analysis found that the weight-loss efficacy profile of 30 mg phentermine was only marginally superior to that of 15 mg phentermine. Thus, the mean 6mo-BWR% was similar for 15 mg phentermine (9.3%) and 30 mg phentermine (10.4%), with only statistically significant differences in a couple of intermediate monthly assessment points between the two treatments. Additionally, a comparable non-cumulative proportion of subjects treated with 15 mg or 30 mg phentermine achieved 6mo-BWR% in the intervals of 5% to <10%, 10% to <15%, and 15% to <20%, with no significant differences, except at the >20% level in favor of 30 mg phentermine. The observed changes in weight loss can be attributed to the administered dose of phentermine, as both dosage groups were balanced regarding the measured anthropometric and clinical variables, with the exception of the percentage of patients with hypertension; however, there is no evidence that hypertension increases or decreases the efficacy of phentermine.

A direct comparison of our findings on the efficacy of phentermine with that reported by other authors is complex, due to differences in efficacy parameters ethnicity, obesity class, treatment duration, comorbidities, and the intermittent or continuous dosing regimens used in the various studies (20–23). However, in a 28-week controlled study evaluating the efficacy of the phentermine and topiramate combination, BWR% of 6.7% and 7.4% and proportions of 43.3% and 46.2% of patients with weight reductions >5% with the 7.5 mg and 15 mg phentermine as monotherapy, respectively, were reported (24). These findings are in line with the marginal difference of phentermine observed in our analysis.

This analysis also confirmed the existence of the previously described large intrasubject variability (13, 15, 25) at all time points with 15 mg and 30 mg phentermine, which highlights the possibility from not losing weight until achieving reductions ≥20% or even greater throughout the treatment. Thus, a number of subjects can achieve significant reductions in body weight using the low dose of 15 mg phentermine, with the safety advantages that this represents.

Considering that multiple studies have shown initial short-term weight loss after starting medication predicts further weight loss, and that tolerance or tachyphylaxis tends to develop after a few weeks of use with phentermine or other drugs, our group has conducted several clinical trials with short-term AOMs, including phentermine 30 mg. The corresponding results led to the proposal that the combination of these two parameters is a good predictor of 6mo-BWR% efficacy. It is important to note that tolerance to phentermine treatment, as assessed in this analysis, does not necessarily imply pharmacological tolerance or tachyphylaxis. Instead, it may result from metabolic adaptation or behavioral factors, such as changes in medication adherence, dietary intake physical activity, among others.

In this study with real-life data, our analysis showed that the single correlation of 1mo-BWRkg or moT against 6mo-BWR% had acceptable Pearson r coefficients for both 15 mg phentermine and 30 mg phentermine. Furthermore, this correlation was notably improved by combining 1mo-BWRkg and moT as predictors of 6mo-BWR%. Likewise, in this analysis, the categorization of patients in various intervals of weight reduction in the first month and development of tolerance, showed similar and increasing levels of 6mo-BWR% induced by 15 mg phentermine and 30 mg phentermine. Thus, the subgroups with 1mo-BWRkg in the <2 kg range, regardless of tolerance development, tended to show 6mo-BWR% ineffectiveness levels (<5%). The 1mo-BWRkg in the 3 to <6 kg subgroup with early tolerance and 1mo-BWRkg 2 to <3 kg subgroups with late tolerance or no tolerance tended to show primarily 6mo-BWR% of 5% to <10%, with a low percentage of cases showing 6mo-BWR% of 10% to <15%. In addition, the subgroup with 1mo-BWRkg ≥6 kg in early tolerance and subgroups 3 to <6 kg and ≥6 kg ranges in late tolerance and no tolerance tended to achieve the best 6mo-BWR% levels of 10% to <15% or more at 6 months.

These efficacy results differ with those obtained by our group, although they were comparable in the intervals >4 kg in early/late tolerance and in the intervals >3 kg for non-tolerant patients. This discrepancy could be due to differences in the design of the studies, since this analysis only included subjects with 6 months of complete treatment (26). Moreover, this analysis has limitations, such as the fact that the data evaluated are from an uncontrolled, open-label study. Additionally, the generalizability of our data is restricted by the relatively small sample size, which is primarily composed of healthy women aged 40 years and older who have class 1 obesity. Furthermore, restricting the analysis to subjects who completed six months of treatment introduces selection bias regarding the overall efficacy of phentermine. On the other hand, it is important to consider that the observed correlation between 1mo-BWRkg + moT and 6mo-BWR% only allows an early associative stratification of the response to phentermine.

Notwithstanding, taken together, our results suggest that once phentermine is selected for initial obesity treatment, it is rational to start with the 15 mg dose of phentermine, given the marginal difference in efficacy, but no of adverse events frequency, compared with 30 mg phentermine (27), and that titration could be relevant on an individual basis. Additionally, it is clear that the administration of 15 mg or 30 mg phentermine is associated with highly variable BWR% values, ranging from negative to greater than 20%. Since individual AOMs efficacy can be defined just in terms of trial and error, an undeniable advantage of 15 mg and 30 mg phentermine is that their acute 6mo-BWR% effectiveness can be predicted early and progressively, based on 1mo-BWRkg and moT, which would also allow for an early decision on whether to continue or modify the treatment according to the individual objective considered. Interestingly, the multivariate analysis revealed that an elevated initial weight correlates with a minor decrease in predicted weight loss. This is a critical finding, considering that class 1 obesity represents the predominant category among Mexican adults with obesity. Furthermore, additional data on the clinical rationale and recent findings regarding the efficacy and potential use of phentermine could provide a comprehensive and contextualized perspective to optimize the use of this drug.

Regarding the general efficacy data related to phentermine use, the first reasonable expectation could be the acquisition of healthy habits through behavioral modification based on diet and exercise. It could be assumed that patients remaining in treatment for 6 months or longer will assimilate the importance of behavioral changes and adopt healthier habits. Beyond the recognition of obesity as a primary risk factor for early mortality, the relevance of lifestyle change is supported by studies showing that adopting 4 consistent habits—consuming ≥5 fruits or vegetables daily, exercising regularly >12 times a month, avoiding excessive alcohol consumption, and not smoking—are associated with a significant decrease in mortality, regardless of initial body mass index (28). Furthermore, there are authors who maintain that the degree of weight loss and its preservation should not be the sole metric of obesity treatment success and that lifestyle changes over the long-term will likely improve the health of patients even in the absence of major weight loss (29). However, it would probably be difficult to convince patients of the success of an obesity treatment without weight loss.

Other relevant efficacy expectation with phentermine use is the potential effective escalation for some non-responders of behavioral treatment. Thus, a recent published trial showed that in obese patients who did not respond to initial behavioral treatment, adding phentermine 15 mg/day for 24 weeks resulted in an additional 5.9% weight loss compared to 2.8% in the placebo-behavioral group (30). In line with this, the expectation of a modest weight reduction (3% to 5%) associated with an improved glycemic control, reduced cardiovascular risk, or improved quality of life; or a greater reduction (10% to 20% or more) for the improvement of non-alcoholic fatty liver disease, sleep apnea, or even aesthetic purposes, may be the goal of obesity treatment (31–33). Although it is clear that the weight-reducing effectiveness of phentermine is variable, an undeniable advantage of 15 mg and 30 mg phentermine is that their early and progressive 6mo-BWR% effectiveness can be reasonably predicted based on weight loss in the 1mo-BWRkg and moT. Since individual AOMs efficacy can be defined just in terms of trial and error, the corresponding early efficacy expectations would also allow for an early decision on whether to continue or modify the treatment according to the individual objective considered.

Once a drug is discontinued, weight regain is the norm. Based on data from treatment with the anorexigenic mazindol for 6 months, it was found that up to approximately 30% of patients maintained the weight loss achieved after 6 months of drug withdrawal, with just the recommendation to follow diet and exercise. It has also been reported that up to 55% of patients treated with phentermine who discontinued the anorectic and started treatment with orlistat (120 mg three times a day) maintained the weight loss achieved after 6 months of drug withdrawal. Additionally, it has been reported an average BWR of 7% after the continued use of phentermine for two years (34). One outstanding issue that deserves further study is whether the early addition of agents such as orlistat and metformin could help to reverse some cases of the acute ineffectiveness of phentermine (BWR% < 5%) in previous non-responders.

On the other hand, in Mexico, several strengths and pharmaceutical forms of phentermine are available for oral administration, including 8 mg, 15 mg, and 30 mg of immediate-release (IR), along with 30 mg of extended-release formulations (ER). Pharmacokinetic data for 30 mg phentermine hydrochloride ER sold in Mexico are not available, as these are not included in the corresponding prescribing information. In addition, it has been reported that the clinical significance of a reduced but prolonged peak concentration in the ER formulations of phentermine is unknown, since plasma phentermine concentrations do not correlate with weight loss effect induced by this agent (35). Moreover, the reported differences in the elimination half-life of phentermine ER against phentermine IR (20 h vs. 8 h, respectively), as well as the persistence of therapeutic concentrations of phentermine for more than 20 h in ER formulations would seem incompatible with the recommendation of daytime administration to avoid insomnia (36).

## Conclusions

Our results suggest that once phentermine is selected for initial obesity treatment, starting with a 15 mg dose is a rational approach. Phentermine can be considered as an agent of moderate efficacy that has shown to be useful for up to 6 months for adopt healthy lifestyle practices, increase life expectancy, augment quality of life, and avoid health hazards in the Mexican population. In addition, phentermine could be a good long-term alternative to the newer, highly effective, and costly AOMs in the Mexican population, taking into account the high prevalence of mainly class 1 obesity, the possibility of effective and safe dose titration, the predictability of early and progressive efficacy after administration of the two 15 mg and 30 mg phentermine strengths, and its affordability. Further controlled studies are needed to establish its long-term efficacy and safety as monotherapy or polytherapy.

## Data Availability

The original contributions presented in the study are included in the article, further inquiries can be directed to the corresponding author.
